# Middle School Effects of the *Dating Matters*® Comprehensive Teen Dating Violence Prevention Model on Physical Violence, Bullying, and Cyberbullying: a Cluster-Randomized Controlled Trial

**DOI:** 10.1007/s11121-019-01071-9

**Published:** 2019-12-12

**Authors:** Alana M. Vivolo-Kantor, Phyllis Holditch Niolon, Lianne Fuino Estefan, Vi Donna Le, Allison J. Tracy, Natasha E. Latzman, Todd D. Little, Kyle M. Lang, Sarah DeGue, Andra Teten Tharp

**Affiliations:** 1 Division of Violence Prevention, National Center for Injury Prevention and Control, Centers for Disease Control and Prevention, 4770 Buford Highway NE, MS-S106-8, Atlanta, GA 30341-3717 USA; 2 Oak Ridge Institute for Science and Education (ORISE), 1299 Bethel Valley Rd, Oak Ridge, TN 37830 USA; 3 2M Research Services, LLC, Arlington, TX USA; 4 Institute for Measurement, Methodology, Analysis and Policy, Texas Tech University, 2500 Broadway, Lubbock, TX 79409 USA

**Keywords:** Violence prevention, Bullying, Cyberbullying, Youth violence, Randomized controlled trial, Dating Matters

## Abstract

Few comprehensive primary prevention approaches for youth have been evaluated for effects on multiple types of violence. *Dating Matters®: Strategies to Promote Healthy Teen Relationships (Dating Matters)* is a comprehensive teen dating violence (TDV) prevention model designed by the Centers for Disease Control and Prevention and evaluated using a longitudinal stratified cluster-randomized controlled trial to determine effectiveness for preventing TDV and promoting healthy relationship behaviors among middle school students. In this study, we examine the prevention effects on secondary outcomes, including victimization and perpetration of physical violence, bullying, and cyberbullying. This study examined the effectiveness of *Dating Matters* compared to a standard-of-care TDV prevention program in 46 middle schools in four high-risk urban communities across the USA. The analytic sample (*N* = 3301; 53% female; 50% Black, non-Hispanic; and 31% Hispanic) consisted of 6th–8th grade students who had an opportunity for exposure to *Dating Matters* in all three grades or the standard-of-care in 8th grade only. Results demonstrated that both male and female students attending schools implementing *Dating Matters* reported 11% less bullying perpetration and 11% less physical violence perpetration than students in comparison schools. Female *Dating Matters* students reported 9% less cyberbullying victimization and 10% less cyberbullying perpetration relative to the standard-of-care. When compared to an existing evidence-based intervention for TDV, *Dating Matters* demonstrated protective effects on physical violence, bullying, and cyberbullying for most groups of students. The *Dating Matters* comprehensive prevention model holds promise for reducing multiple forms of violence among middle school-aged youth. ClinicalTrials.gov Identifier: NCT01672541

**Electronic supplementary material:**

The online version of this article (10.1007/s11121-019-01071-9) contains supplementary material, which is available to authorized users.

Over the past few decades, violence has been identified as a public health problem impacting individuals, communities, and society as a whole (Mercy et al. 2002). Estimates from the 2017 Centers for Disease Control and Prevention’s (CDC) national Youth Risk Behavior Survey (YRBS) indicate that almost 24% of high school students reported being in a physical fight, 19% reported being bullied in person on school property, and 15% reporting being bullied electronically in the past 12 months (Kann et al. 2018). Experiencing youth violence can have severe and lasting consequences for both victims and perpetrators; victims of youth violence report decreases in self-esteem, increases in psychological trauma, and avoiding school and skipping classes (Esbensen and Carson 2009; Hertz et al. 2015; Rigby 2003; Song et al. 1998).

## The Overlap of Multiple Forms of Violence

Emerging evidence points to the overlap of multiple forms of youth violence including physical fighting, bullying, sexual violence, and teen dating violence (TDV). Previous studies have found that poly-victimization, or exposure to multiple types of violence (e.g., sexual abuse, bullying), is common among children ages 2–17 (Turner et al. 2010) and that poly-victimization is predicted by delinquency and aggression (Finkelhor et al. 2007; Margolin et al. 2010). In addition, engagement in violence is a strong predictor of future violent behaviors. For example, youth who are both victims and perpetrators of bullying are at an increased risk for delinquency and violence over time (Ttofi et al. 2012). Research from Bender and Lösel (2011) demonstrated that self-reported physical bullying at age 14 was a significant predictor of delinquency, violent offending, drug use, and aggressive behavior approximately 10 years later in young adulthood.

More recently, several studies have demonstrated a longitudinal relationship between bullying, sexual harassment, and TDV. The *Bully-Sexual Violence Pathway* theory (Espelage et al. 2012) suggests that youth who perpetrate bullying in early adolescence may be at greater risk than their peers for perpetrating sexual violence in late adolescence. This theory was empirically supported by Espelage et al. (2015); males who engaged in bullying behaviors in 5th grade were more likely to perpetrate sexual harassment in 7th grade when they also participated in homophobic name-calling with peers in 5th grade. In addition, Foshee et al. (2014a) conducted one of the first studies to develop the longitudinal association between bullying perpetration and TDV perpetration. In a sample of middle school students, self-reported in-person, physical bullying perpetration in 6th grade was a significant predictor of the onset of physical dating violence perpetration in 8th grade. A further study extended these findings to specify that bullying perpetration predicted TDV perpetration only among students who did not also report bullying victimization (Foshee et al. 2016a).

## Cross-Cutting Prevention Approaches

In the past, prevention programs have often been developed and implemented to address a single problem or risk behavior—for example, communities may implement one program to prevent bullying and a different program to prevent TDV (Miller et al. 2012; Olweus 2005). However, given evidence that multiple forms of violence often overlap, it is important to consider why this overlap occurs in order to inform prevention approaches. One idea is that several forms of violence share common risk and protective factors, thus making it possible to conceive that a single program addressing common risk factors and/or promoting common protective factors may prevent multiple forms of violence. Foshee et al. (2016b) found that poor conflict management skills, acceptance of TDV, low maternal responsiveness, and poor mother-adolescent communication were the most important shared risk factors across bullying, sexual harassment, and physical TDV. In addition, there is evidence these behaviors share common protective factors including family connectedness, association with prosocial peers, and strong connection/commitment to school (Capaldi et al. 2012; Elgar et al. 2009; Hong and Espelage 2012; Salmivalli et al. 1997).

Given this and other research demonstrating shared risk and protective factors for violence, there have been increasing calls for cross-cutting prevention strategies, including programs that prevent multiple forms of youth violence and other adolescent risk behaviors (CDC 2016). Although cross-cutting middle school prevention programs effective at preventing multiple risk behaviors are limited, there are a few examples to note. *LifeSkills Training* (LST), a school-based substance abuse prevention program, not only reduces substance use/abuse (Botvin et al. 2001) but also other risk behaviors not directly targeted, such as risky driving (Griffin et al. 2004), verbal and physical aggression, fighting, and delinquency among adolescents (Botvin et al. 2006). *Safe Dates* is a dating violence prevention program that has demonstrated reductions in physical and sexual dating violence victimization and perpetration, peer violence victimization and perpetration, and weapon carrying (Foshee et al. 2004; Foshee et al. 2014b). Finally, *Fourth R* is a school-based program aimed at reducing violence, substance abuse, and unsafe sex in adolescence. A cluster-randomized trial demonstrated *Fourth R* reduced physical dating violence, reduced violent delinquency, and increased condom use in the intervention group over a 2.5-year follow-up, relative to the control group (Wolfe et al. 2009; Crooks et al. 2011). Although additional research is needed, these studies suggest that prevention approaches addressing shared risk and protective factors may be an efficient and effective prevention strategy for resource-strapped schools and communities. Evidence is growing that some prevention programs targeting multiple risk and protective factors (i.e., LST) have economic benefits that exceed implementation costs (Washington State Institute for Public Policy 2018). That said, these programs focus mostly on the inner levels of the social ecology—individual- and relationship-level—and do not target risk and protective factors or context at the outer levels of the social ecology such as school and community climate.

## The *Dating Matters* Comprehensive Teen Dating Violence Prevention Model

To address the need for effective TDV prevention strategies to impact multiple forms of violence in adolescence, CDC developed *Dating Matters®: Strategies to Promote Healthy Teen Relationship* (*Dating Matters*; www.cdc.gov/violenceprevention/datingmatters). The *Dating Matters* comprehensive TDV prevention model is focused on the primary prevention of TDV and the promotion of respectful relationship behaviors, as well as the prevention of other risk behaviors, including other forms of violence, among middle school students (Tharp et al. 2011; Tharp 2012; Niolon et al. 2016, 2019). *Dating Matters* moves beyond prior single-program interventions to include multiple preventive components at the individual, relationship, and community levels of the social ecology, including (1) classroom-delivered programs for 6th, 7th, and 8th grade students; (2) training for parents of 6th, 7th, and 8th grade students; (3) training for educators; (4) a youth communications program; and (5) activities at the local health department to build prevention capacity and track TDV-related data and policies. In its promotion of healthy, respectful relationships and its provision of multiple opportunities for developing skills such as conflict management and communication, *Dating Matters* addresses a constellation of risk and protective factors that can prevent multiple forms of violence.

An effectiveness evaluation of *Dating Matters* demonstrated significant and positive preventive effects on TDV behaviors and negative conflict styles among 6th–8th graders when compared to an evidence-based standard-of-care program (*Safe Dates*) (Niolon et al. 2019). Although promoting healthy relationships and preventing TDV are the primary intervention outcomes, the content and skills-based activities target many risk and protective factors for TDV, including youth violence and delinquency (e.g., fighting, bullying, cyberbullying, etc.), sexual risk-taking behaviors, sexual harassment, and substance use based on learning approaches (e.g., cognitive-behavioral, social learning) that have been shown to effectively influence health-related and problem behaviors among youth (Estefan et al. 2019; Greenberg et al. 2001; Kazdin and Weisz 1998). For example, a session in the both the 6th and 7th grade youth programs teaches skills for emotional regulation and promotes emotional literacy, or the ability to identify, understand, and respond to feelings in a healthy and safe way. Such skills are common protective factors for multiple forms of violence and can transcend the prevention of TDV behaviors and prevent other co-occurring forms of violence among youth as well.

## The Present Study

This paper describes the results of a comparative effectiveness cluster-randomized controlled trial evaluating effects of the *Dating Matters* comprehensive prevention model compared to a standard-of-care intervention on secondary outcomes among two cohorts of students who were in 6th–8th grades during the implementation phase of the trial and were therefore eligible for full exposure to *Dating Matters*. We hypothesized that the *Dating Matters* comprehensive model would be significantly more effective at preventing perpetration and victimization of physical fighting, bullying, and cyberbullying compared to *Safe Dates*.

## Methods

### Design and Participants

A multi-site, cluster-randomized controlled trial was conducted with 46 middle schools in four high-risk, urban sites across the USA. Specifically, sites were selected based on elevated levels of both violent crime (e.g., homicide, aggravated assault, felony assault, sexual assault) *and* economic hardship (i.e., poverty). Participating schools were randomized prior to survey data collection to receive the *Dating Matters* comprehensive prevention model (DM) in 6th–8th grades or a standard-of-care (SC) TDV prevention program *Safe Dates* (Foshee et al. 2004), in 8th grade only. Following randomization, schools worked with local health departments at each site and CDC contract staff to obtain parental permission. With parental consent in place, 6th–8th grade students in both the DM and SC middle schools were approached to participate in biannual surveys over four school years (fall 2012–spring 2016) for a total of six surveys in middle school. Informed assent was obtained from all participants prior to completing any survey. The overall survey participation rate was 79.7%. Additional information about the *Dating Matters* study, including recruitment, implementation, data collection procedures, and the CONSORT study diagram, are available elsewhere (Niolon et al. 2016, 2019). All procedures and materials were approved by multiple Institutional Review Boards.

For the current analysis, we included schools that had implemented either DM (*N* = 22) or SC (*N* = 24) for at least two full academic years. The analyzed sample of 3301 students (DM: *N* = 1662; SC: *N* = 1639) includes two full-exposure cohorts (i.e., cohorts 3 and 4 that had the potential to receive all 3 years of intervention components in the DM condition). The mean age was 11.93 years in the fall semester of 6th grade (SD = 0.57) with slightly more females (*N* = 1750; 53%) than males (*N* = 1551; 47%). The sample was predominantly Black, non-Hispanic (*N* = 1641, 50%) and Hispanic (*N* = 1022, 31%). Less than 1% were Native American/Alaskan native or Native Hawaiian/other Pacific Islander. See Table 1 for all sample socio-demographics.

**Table 1:** Demographic breakdown of total sample by sex, cohort, and treatment

	Females—Cohort 3		Females—Cohort 4		Males—Cohort 3		Males—Cohort 4	
	SC (*N* = 428)	DM (*N* = 444)	SC (*N* = 418)	DM (*N* = 460)	SC (*N* = 401)	DM (*N* = 399)	SC (*N* = 392)	DM (*N* = 359)
	*p*	*p*	*p*	*p*	*p*	*p*	*p*	*p*
White, nh	1.9%	5.2%^a^	2.9%	6.1%^a^	1.7%	5.0%^a^	4.8%	5.3%
Black, nh	48.6%	50.0%	49.0%	49.8%	48.6%	54.9%^a^	48.7%	47.9%
Pacific Islander, nh	0.9%	0.7%^a^	0.0%	0.4%	0.0%	0.8%	0.8%	0.3%^a^
Asian, nh	6.8%	4.7%^a^	8.4%	7.4%	6.0%	4.0%^a^	10.5%	9.2%
Native American, nh	1.2%	1.1%	0.0%	0.2%	1.0%	0.5%^a^	0.8%	0.3%^a^
Mixed, nh	10.0%	8.8%	6.9%	7.6%	4.2%	5.3%^a^	3.3%	9.7%^a^
Hispanic	30.6%	29.5%	32.8%	28.5%^a^	38.4%	29.6%^a^	31.1%	27.3%^a^
	*M* (*s*^2^)	*M* (*s*^2^)	*M* (*s*^2^)	*M* (*s*^2^)	*M* (*s*^2^)	*M* (*s*^2^)	*M* (*s*^2^)	*M* (*s*^2^)
Age	11.87 (0.58)	11.91 (0.53)	11.87 (0.55)	11.88 (0.53)	11.97 (0.56)	11.99 (0.59)	11.94 (0.59)	12.00 (0.65)

*SC* standard-of-care condition, *DM* Dating Matters condition, *p* proportion, *M* mean, *s*^*2*^ variance, *nh* non-Hispanic

^a^Flags baseline inequivalence (Cox or Hedge’s *g* test statistic > 0.05)

### Measures

#### Bullying

Bullying victimization and perpetration were measured using selected items from the Illinois Bully Scale (Espelage and Holt 2001) at baseline and subsequent follow-up surveys. Students were asked, “In the last 30 days at school, how often did these things happen…” and were provided six items on perpetration behaviors (e.g., I upset other students for the fun of it, I helped harass other students, I teased other students), and two items on victimization (e.g., Other students called me names). All items referenced behaviors that happened in person rather than online, and had response options: 1 = never, 2 = 1 or 2 times, 3 = 3 or 4 times, and 4 = 5 or more times. Three parceled latent indicators were created from the six perpetration items (for details on the process used for selecting and constructing parcels, see Niolon et al. 2019). Cronbach’s alpha ranged from .78 to .81 across time for perpetration items. The correlation between the two victimization items ranged from .79 to .84 across time. The raw means for composites ranged from 1.17 to 1.32 for perpetration and 1.45 to 1.82 for victimization across time and sex by condition.

#### Cyberbullying

Cyberbullying was measured with a total of four items from the AAUW Sexual Harassment Survey (American Association of University Women Educational Foundation 2001)—two victimization items and two perpetration items. At baseline, students were asked “In your lifetime, how often, if at all, did someone do the following things to you/did you do the following to others?” At follow-up survey time points, the recall period was “past four months.” Items included, for example, “someone spread rumors about me online, whether they were true or not” and “I made aggressive or threatening comments to anyone online.” All items had the response options: 1 = never, 2 = 1 to 3 times, 3 = 4 to 9 times, and 4 = 10 or more times. The raw means for composites of perpetration ranged from 1.05 to 1.14 and victimization ranged from 1.10 to 1.22 across time and groups. The correlation between the perpetration items ranged from .46 to .57 and between the victimization items ranged from .21 to .54 across time and sex by condition.

#### Physical Violence

Physical violence was assessed with two items: “In the past six months (baseline)/four months (follow-up), how often did you attack someone with the idea of seriously hurting them?” and “…get attacked by someone who seemed to want to seriously hurt you?” Both items had the response options: 1 = never, 2 = 1 or 2 times, 3 = 3 or 4 times, and 4 = 5 or more times. The raw means for the physical violence perpetration item and the physical violence victimization item across time and sex by condition ranged from 1.04 to 1.21 and 1.05 to 1.21, respectively.

### Analysis Plan

Before analyses were conducted, we conducted baseline equivalence testing, employed multiple imputation of missing data using PcAux (Lang and Little 2018), and created 100 imputed datasets (see eMethods in Niolon et al. 2019). Our imputation process was conducted under the assumption of missing at random for the outcome variables and demographics. School dropout and replacement resulted in an average of 7% missing data in the student-level outcome scores, and within participating schools, entry and exit of students (e.g., transfer students, opt out) resulted in an average of 43% missing data that ranged from 30 to 55% across all waves. Among students who took the survey, item non-response accounted for an average of 17% for bullying items, 14% of cyberbullying items, and 19% for physical violence items. Data for students in cohorts 3 or 4 with participation in at least one out of the six surveys used in this study were imputed. All descriptive statistics based on a single grand mean imputed dataset and all models were run on 100 imputed datasets. We used a standardization process for each outcome indicator to reflect a “percent of maximum score” or POMS (Little 2013). This procedure rescaled the original response option metric of the multiple item composite (average score across items) to range from 0 to 100 using the equation ((*X* − 1)/4) × 100. For example, a full set of “never” responses would receive a 0 and a full set of responses reflecting “5 or more times” (bullying, physical violence) or “10 or more times” (cyberbullying) would receive a score of 100.

Outcome variables were adjusted for covariates including the following: school (clustered design effect); recall timeframe for behaviors; witnessing violence in the community and home; relative age within grade; race/ethnicity; guardian status (e.g., living in single parent household); and lag in assessment timing from one survey to the next (Niolon et al. 2019). We conducted multiple group (i.e., sex by cohort by treatment) structural equation modeling using Mplus Version 7.4 (Muthén and Muthén 2012). In separate models for the six outcomes, statistically equivalent means across the groups and time points were constrained to be equal such that non-significant differences at *p* < .01 appear as overlapping lines. Chi-square difference tests of overall fit were used to evaluate the number and composition of constraints and post hoc Wald *χ*^2^ tests determined if the resulting constrained means were statistically distinguishable from one another using strict criteria of evaluating model fit (*p* > .2). This approach simplifies the model, imposing parsimony and decreasing the likelihood of Type II errors (“false negatives”; Little and Lopez 1997). The magnitude of prevention effects is estimated as the difference between DM and SC students in constrained POMS scores at each time point and as the risk of each outcome in the DM group, relative to SC within sex and cohort.

## Results

**Fig. 1:**
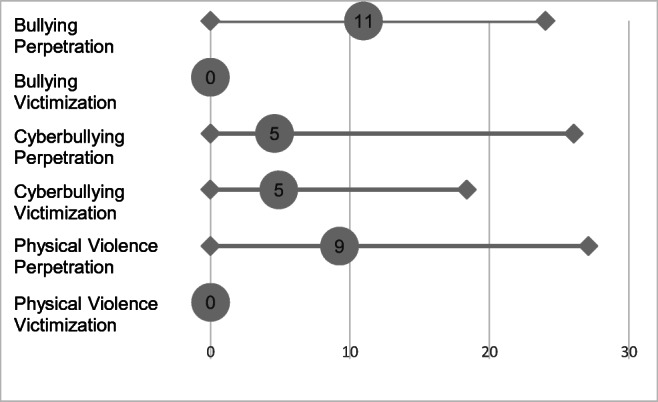
Percent relative risk reduction by outcome (M, range) for Dating Matters vs. standard-of-care. Note: Relative risk reduction represents the percent reduction in scores on measures of victimization and perpetration of bullying, cyberbullying, and physical fighting for the Dating Matters condition relative to the standard-of-care condition. The numbers within the circles represent the average risk reduction for that outcome across the 4 groups (sex by cohort), and the space between the diamonds represent the range of relative risk reduction on that outcome across the four groups

Baseline equivalence across DM and SC conditions indicated some evidence of equivalence with respect to age at baseline, there were more Hispanic and fewer non-Hispanic White and non-Hispanic Black students in schools assigned to the SC condition (Table 1). All outcomes passed baseline equivalence tests. Figure 1 provides the percent relative risk reduction by outcome for DM compared to the SC for all outcomes; however, below we provide additional results by sex for each outcome.

### Bullying Perpetration and Victimization

**Fig. 2:**
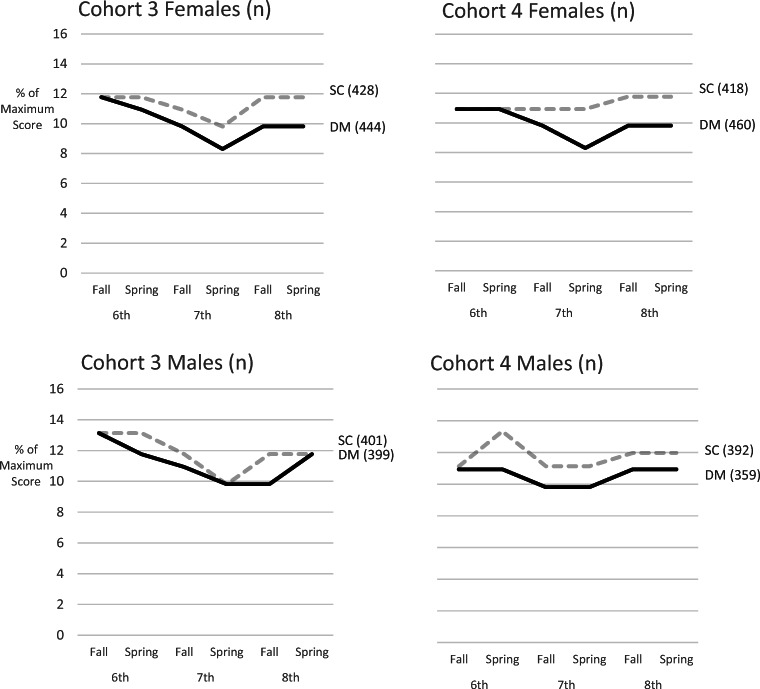
Bullying perpetration across time by sex and cohort. Note: SC = standard-of-care condition; DM = Dating Matters condition. Percent of Maximum Score (POMS) refers to the maximum possible score given the number of items and response categories in a scale, rather than the maximum observed score. Mean POMS scores have been constrained to be equal when not significantly different; non-overlapping lines at any time point represent a statistically significant group difference

The constrained means for bullying perpetration are shown in Fig. 2. The differences between DM and SC students on model-constrained bullying perpetration scores averaged 1.27 POMS (range = 0.00–2.63) across sex/cohort groups, and the direction of all significant differences was consistent with protective intervention effects (i.e., DM students have lower average scores than their SC counterparts). These results revealed that, on average, students in DM schools scored 11% lower than students in SC schools on the measure of bullying perpetration (range: 0–24%). The average relative risk reduction in bullying perpetration scores for DM females was 13% (range = 0.00–24.03) and 9% for DM males (range = 0.00–16.77) compared to their SC counterparts. No effects were found for bullying victimization

.

### Cyberbullying Perpetration and Victimization

**Fig. 3:**
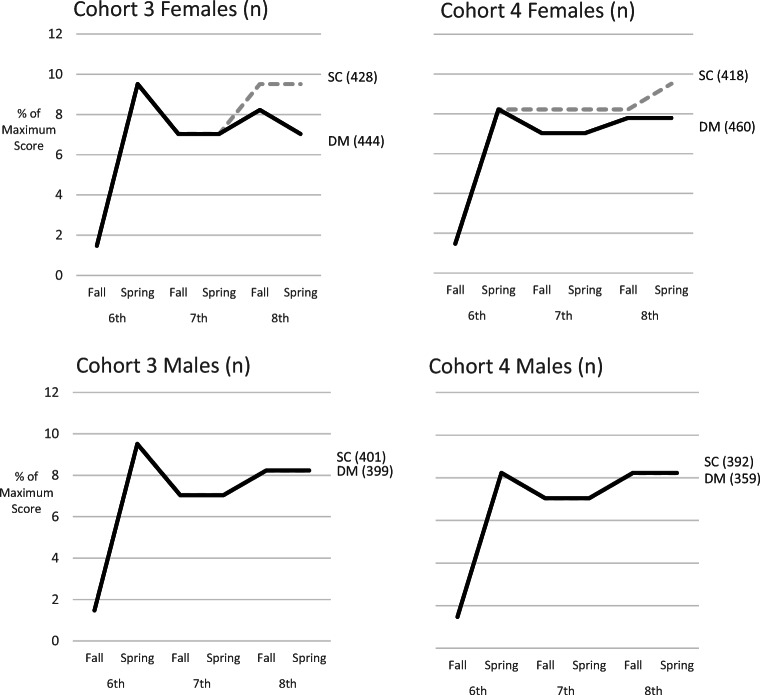
Cyberbullying perpetration across time by sex and cohort. Note: SC = standard-of-care condition; DM = Dating Matters condition. Percent of Maximum Score (POMS) refers to the maximum possible score given the number of items and response categories in a scale, rather than the maximum observed score. Mean POMS scores have been constrained to be equal when not significantly different; non-overlapping lines at any time point represent a statistically significant group difference

**Fig. 4:**
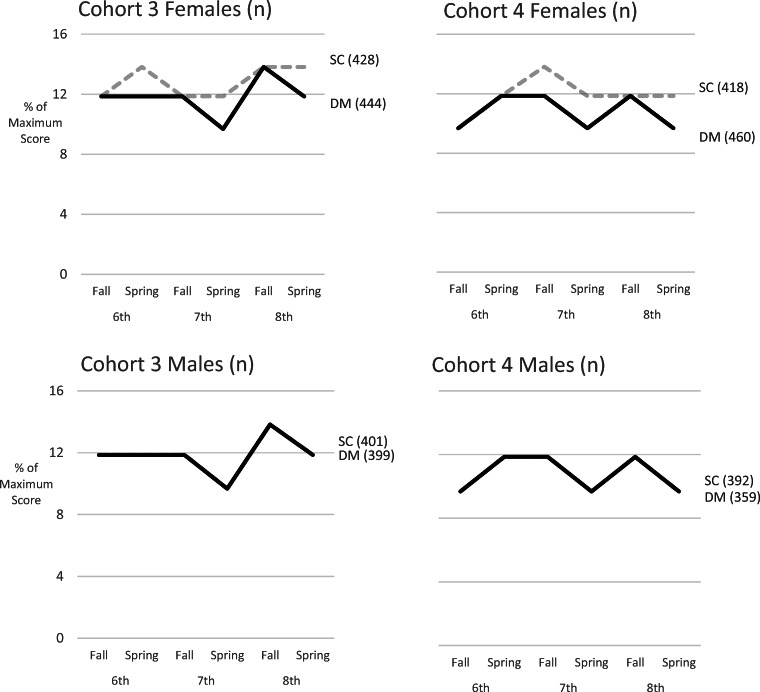
Cyberbullying victimization across time by sex and cohort. Note: SC = Standard-of-care condition; DM = Dating Matters condition. Percent of Maximum Score (POMS) refers to the maximum possible score given the number of items and response categories in a scale, rather than the maximum observed score. Mean POMS scores have been constrained to be equal when not significantly different; non-overlapping lines at any time point represent a statistically significant group difference

The constrained means for cyberbullying perpetration and victimization are presented in Figs. 3 and 4. The differences between DM and SC students’ cyberbullying perpetration scores averaged 0.42 POMS (range = 0.00–2.48); differences in cyberbullying victimization scores averaged 0.62 POMS (range = 0.00–2.18). While there were no significant program effects for males, we found program effects for females. The average relative risk reduction in cyberbullying perpetration scores for DM females was 9% (range = 0–26%), and for cyberbullying victimization scores were 10% lower (range = 0–18%) than for SC females.

### Physical Violence Perpetration and Victimization

**Fig. 5:**
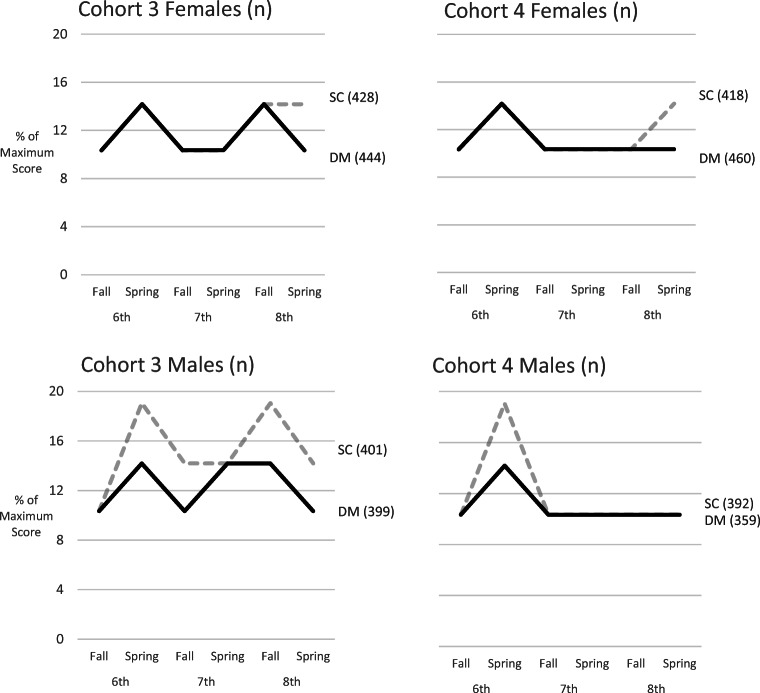
Physical violence perpetration across time by sex and cohort. Note: SC = standard-of-care condition; DM = Dating Matters condition. Percent of Maximum Score (POMS) refers to the maximum possible score given the number of items and response categories in a scale, rather than the maximum observed score. Mean POMS scores have been constrained to be equal when not significantly different; non-overlapping lines at any time point represent a statistically significant group difference

The constrained means for the physical violence perpetration are shown in Fig. 5. The differences between DM and SC students on physical violence perpetration scores averaged 1.50 POMS (range = 0.00–4.87). These results showed that students attending DM schools scored 9% lower on average on the measure of physical violence perpetration than students attending SC schools (range: 0–27%). We found differences consistent with hypothesized intervention effects for males (average 13%, range = 0.00–27.09) and females (average 5%, range = 0.00–27.09). One notable exception to the consistent pattern of program effects was that Cohort 4 males demonstrated program effects on physical violence perpetration at spring of 6th grade only; DM and SC male students in that cohort were not significantly different on physical violence perpetration in 7th and 8th grade. No effects were found for physical violence victimization (Fig. 5).

## Discussion

The results demonstrate that when compared to another evidence-based TDV prevention program, the *Dating Matters* comprehensive prevention model can be effective at preventing physical violence, bullying, and cyberbullying in middle school. Overall, male and female students at *Dating Matters* schools scored significantly lower on measures of bullying perpetration and physical violence perpetration, and female students scored significantly lower on cyberbullying perpetration and victimization as compared to students at schools implementing standard-of-care program. The *Dating Matters* model, which encourages respectful treatment of others, not just dating partners, through social-emotional learning may provide students the opportunity to learn about healthy relationships and practice the skills necessary to negotiate and maintain healthy relationships through respecting others and themselves. In addition, repeated exposure to these lessons over the 3 years of middle school, and in the context of other school and community prevention activities that are part of the comprehensive *Dating Matters* intervention, may have boosted its effectiveness beyond potential effects of the existing evidence-based TDV program implemented only in the 8th grade.

CDC’s *Preventing Multiple Forms of Violence: A Strategic Vision for Connecting the Dots* (CDC 2016) suggests that preventing multiple forms of violence can be accomplished by addressing common risk and protective factors. These results demonstrate that *Dating Matters* addresses multiple forms of interpersonal violence beyond TDV. We suggest that this may be the case because *Dating Matters* was designed to address risk and protective factors associated with multiple forms of interpersonal violence by reducing risky conflict management styles and improving social-emotional skills. Additionally, to our knowledge, it is the only comprehensive TDV prevention model to incorporate complementary prevention strategies across the individual, relationship, and community levels of the social ecology. Results from this study support the idea that prevention strategies addressing concepts relevant to multiple adolescent risk behaviors, such as the importance of healthy and respectful relationships and building skills to engage in them, may prevent or reduce the incidence of multiple forms of violence and aggression. In doing so, this expands the reach of prevention efforts while simultaneously conserving resources for schools and communities.

The specific exceptions to the intervention effects on these outcomes deserve further examination. Effects were found for both females and males in both cohorts of students on in-person bullying and physical violence *perpetration*, but not on in-person bullying or physical violence *victimization*. The reduction in the incidence of perpetration is very encouraging, given that true prevention of any form of violence occurs when violence is stopped at the source with the perpetrator. However, it is interesting, given that *Dating Matters* was implemented with a whole-school approach in neighborhood schools, that victimization was not similarly affected. This could be due to students experiencing bullying and physical violence in numerous contexts in their lives beyond just the school, given their exposure to other kids in their neighborhoods, spiritual communities, and families who might not have been exposed to the intervention. That said, the reduction of incidence of perpetration among all participants is a very promising finding and indicates that youth exposed to *Dating Matters* are being less aggressive and more respectful toward their peers as well as their dating partners (Niolon et al. 2019).

Another exception is that intervention effects were found for cyberbullying victimization and perpetration only among females. It is possible that the measurement of cyberbullying was not sensitive enough to capture the range of behaviors that can fall under this form of aggression, as the measure included only two items. Still, the *Dating Matters* comprehensive model was more effective in preventing cyberbullying among females than the standard-of-care program, and future research should continue to examine effects on both males and females.

Though the overall findings of this study are promising, the study has several limitations to consider. Attempting to conduct a cluster-randomized trial in high-risk urban neighborhoods introduced a number of challenges such as follows: school closures, probation, and dropout; variability within conditions across sites on implementation and on evaluation protocols (e.g., teacher vs. community-based program facilitators); challenges in obtaining parental consent for research participation; and student mobility over time (Niolon et al. 2016). Our reliance on self-report of behaviors across outcomes potentially introduces recall and/or social desirability bias; future studies would benefit from inclusion of observational methods or teacher reports of student behavior. Our measurement may not be precise. For example, physical violence victimization and perpetration were each assessed with one item, and cyberbullying victimization and perpetration were each assessed with two items. In addition, the physical violence victimization and perpetration item did not specifically indicate whether or not students reported violence toward a peer or dating partner. Finally, we conducted this trial with middle school youth in four high-risk, urban areas with above average rates of crime and poverty, and it is not known how generalizable the findings are to other populations.

Despite these limitations, this study has many important strengths. Comprehensive prevention initiatives that can demonstrate effects on multiple forms of violence above and beyond evidence-based single-program interventions may be time- and resource-efficient options for communities facing ever-growing research challenges. Further, despite challenges in implementing this design, the use of a multi-site, cluster-randomized controlled trial to evaluate the *Dating Matters* comprehensive model provides a rigorous test of prevention effects. The comparative effectiveness design makes practical sense in that it provides evidence of effects above and beyond the existing evidence-based prevention program *Safe Dates* implemented with 8th grade students only. Finally, our study was sufficiently powered. Despite low base rates for interpersonal violence victimization and perpetration in this early developmental period, we were able to detect small but significant positive program effects. Future research examining the longer-term outcomes through high school, when interpersonal aggression rates typically increase (Kann et al. 2018), is needed to understand the full potential of investment in a comprehensive prevention model for the prevention of multiple forms of violence.

## Electronic supplementary material

ESM 1 (PDF 83 kb)
