# Correction to: Middle School Effects of the *Dating Matters®* Comprehensive Teen Dating Violence Prevention Model on Physical Violence, Bullying, and Cyberbullying: A Cluster-Randomized Controlled Trial

**DOI:** 10.1007/s11121-020-01113-7

**Published:** 2020-04-14

**Authors:** Alana M. Vivolo-Kantor, Phyllis Holditch Niolon, Lianne Fuino Estefan, Vi Donna Le, Allison J. Tracy, Natasha E. Latzman, Todd D. Little, Kyle M. Lang, Sarah DeGue, Andra Teten Tharp

**Affiliations:** 1 Division of Violence Prevention, National Center for Injury Prevention and Control, Centers for Disease Control and Prevention, 4770 Buford Highway NE, MS-S106-8, Atlanta, GA 30341-3717 USA; 2 Oak Ridge Institute for Science and Education (ORISE), 1299 Bethel Valley Rd, Oak Ridge, TN 37830 USA; 3 2M Research Services, LLC, Arlington, TX USA; 4 Institute for Measurement, Methodology, Analysis and Policy, Texas Tech University, 2500 Broadway, Lubbock, TX 79409 USA

**Correction to: Prevention Science**

10.1007/s11121-019-01071-9

The article “Middle School Effects of the Dating Matters® Comprehensive Teen Dating Violence Prevention Model on Physical Violence, Bullying, and Cyberbullying: a Cluster-Randomized Controlled Trial”, written by Alana M. Vivolo-Kantor, Phyllis Holditch Niolon, Lianne Fuino Estefan, Vi Donna Le, Allison J. Tracy, Natasha E. Latzman, Todd D. Little, Kyle M. Lang, Sarah DeGue, and Andra Teten Tharp, was originally published electronically on the publisher’s internet portal on 12 December 2019 without open access.

With the author(s)’ decision to opt for Open Choice the copyright of the article changed on March 2020 to © The Author(s) 2020 and the article is forthwith distributed under a Creative Commons Attribution 4.0 International License (https://creativecommons.org/licenses/by/4.0/), which permits use, sharing, adaptation, distribution and reproduction in any medium or format, as long as you give appropriate credit to the original author(s) and the source, provide a link to the Creative Commons license, and indicate if changes were made.

